# Surgical Treatment of Chondrosarcoma of the Sternum

**DOI:** 10.1080/13577140120099209

**Published:** 2001-12

**Authors:** Robert U. Ashford, Jeremy Stanton, Farid Khan, Jean A. S. Pringle, Stephen R. Cannon, Timothy W. R. Briggs

**Affiliations:** 1 Hull Royal Infirmary Hull UK; 2 Colchester General Hospital Colchester UK; 3 Queen Elizabeth Hospital Woolwich UK; 4 The London Bone and Soft Tissue Tumour Service The Royal National Orthopaedic Hospital Brockley Hill, Stanmore Middlesex MA7 4LP UK

## Abstract

*Purpose:* We reviewed all tumours of the sternum referred to The London Bone and Soft Tissue Tumour Service between 1956 and 1997 inclusive.

*Patients and results:* There were eight patients with this pathology, the male to female ratio was 3:1 and their mean age was 53 years. Of these patients, three are alive and disease free, one is alive with recurrence, and four have died, two of the consequences of the disease and two of unrelated causes. Surgery is the principal treatment of these tumours both for excision and subsequent reconstruction.

*Discussion:* Extended disease-free survival is possible with correct diagnosis, complete excision at the first operation, appropriate skeletal reconstruction, adequate skin cover and appropriate postoperative support and follow-up.


*Sarcoma* (2001) **5**, 209–213

**ORIGINAL ARTICLE**

### Surgical treatment of chondrosarcoma of the sternum

ROBERT U. ASHFORD[1], JEREMY STANTON[2], FARID KHAN[3], JEAN A.S. PRINGLE[4], STEPHEN R. CANNON[4] & TIMOTHY W.R. BRIGGS[4]

[1]*Hull Royal Infirmary, Hull,* [2]*Colchester General Hospital, Colchester,* [3]*Queen Elizabeth Hospital, Woolwich,* [4]*The London Bone and Soft Tissue Tumour Service, The Royal National Orthopaedic Hospital, Brockley Hill, Stanmore, Middlesex, UK*

**Abstract**

*Purpose:* We reviewed all tumours of the sternum referred to The London Bone and Soft Tissue Tumour Service between 1956 and 1997 inclusive.

*Patients and results:* There were eight patients with this pathology, the male to female ratio was 3:1 and their mean age was 53 years. Of these patients, three are alive and disease free, one is alive with recurrence, and four have died, two of the consequences of the disease and two of unrelated causes. Surgery is the principal treatment of these tumours both for excision and subsequent reconstruction.

*Discussion:* Extended disease-free survival is possible with correct diagnosis, complete excision at the first operation, appropriate skeletal reconstruction, adequate skin cover and appropriate postoperative support and follow-up.

### Introduction

The sternum is an unusual site for malignant bone tumours. A retrospective study of the London Bone Tumour Registry revealed 11 were primary bone tumours of the sternum (excluding bone marrow derived tumours) between 1956 and 1997 inclusive. The majority of the primary bone tumours of the sternum (eight of 11) were chondrosarcomas. The results of surgical treatment of sternal chondrosarcomas are dependent on both grade of tumour and adequacy of resection.

### Patients and methods

Eight patients with chondrosarcomas of the sternum were registered with The London Bone and Soft Tissue Tumour Service. Data was collected by reviewing patients' clinical and operative notes and the histology report from each tumour (Table 1).

Each tumour was graded using the system according to Enneking (Musculoskeletal Tumour Society).[1] Records were assessed with respect to the age and sex of each patient, the symptoms experienced, the tumour grade, definitive treatment, reconstruction of both bone and soft tissue and tumour recurrence.

The mean age was 53 years (range 25–79), with six males and two females. No patients had evidence of metastases at the time of diagnosis. There were a total of 12 surgical procedures (excluding biopsies) to the sternum with all patients having at least one (Table 2). The follow up ranged from 3 to 21 years.

The assessment, management and follow-up of patients over a period of four decades will have varied. We outline our current management protocol later in this paper. However, despite variations over the years, following diagnosis, tumour resection was undertaken in all cases.

### Results

Following radiological imaging, the diagnosis was made by biopsy, three by needle biopsy and five by open biopsy. In all cases, the biopsy diagnosis was subsequently confirmed by histological analysis of the resection specimen. All chondrosarcomas were reported histologically as low grade. The tumours were in the manubrium in two patients and in the body in the other six patients. One patient (patient 7) had an associated pathological condition, Ollier's disease. Enneking (MSTS)[1] staging of the tumours was G1 for patient 7 and G2 for the remainder. The sternum is classified as an extracompartmental site for tumour in the MSTS system[1] and therefore in all patients the tumour site was T2. No patients had metastases at diagnosis (M0). This system stages patient 7 as IB and the remainder as IIB (Table 1).

Correspondence to: T.W.R. Briggs, Royal National Hospital, Brockley Hill, Stanmore, Middlesex MA7 4LP, UK. Tel: 020 8954 2300.

1357–714X print/1369–1643 online/01/040209–05 © 2001 Taylor & Francis Ltd
DOI: 10.1080/13577140120099209

**Table 1.** *Patient data*

| Case | Year | Age | Sex | Biopsy | Histology grade | Stage | Presenting complaint | Time (symptoms to referral) | Follow-up time period | Follow-up status |
|---|---|---|---|---|---|---|---|---|---|---|
| 1 | 1972 | 68 | M | O | 1 | IIB | Enlarging lump | 1 year | 17 years | DUC |
| 2 | 1978 | 26 | F | O | 1 | IIB | | | 21 years | ADF |
| 3 | 1982 | 25 | M | O | 1 | IIB | | | 17 years | ADF |
| 4 | 1983 | 79 | M | O | 1 | IIB | | | 1 year | DUC |
| 5 | 1990 | 73 | M | N | 1 | IIB | Painless Lump | 4 years | 4 years | DoD |
| 6 | 1993 | 67 | M | N | 1 | IIB | Lump – Intermittent Pain | 4 years | 2 years | DoD |
| 7 | 1996 | 58 | F | N | 1–2 | IB | Pain | 9 months | 3 years | ADF |
| 8 | 1996 | 28 | M | O | 1 | IIB | Pain, lump | 3 years 6 months | 3 years | AWD |

O ~ open biopsy N ~ needle biopsy
DoD ~ died of disease DUC ~ died of unrelated cause AWD ~ alive with disease ADF ~ alive and disease free.

**Table 2.** *Treatment*

| Case | Site | Primary treatment | Reconstruction | Soft tissue treatment | Local recurrence | Treatment | Metastases | Treatment |
|---|---|---|---|---|---|---|---|---|
| 1 | Manubrium | Excision | | Primary closure | | | | |
| 2 | Body | Incomplete excision* | Titanium plate | Primary closure | 1979 | Subtotal sternectomy | 1. Diaphragm<br>2. Posterior thoracic | 1. Metastatectomy 1986<br>2. Metastatectomy 1994 |
| 3 | Body | Excision* | Pectoralis major flap | Primary closure | 1984<br>1986 | Wide excision,<br>Radical excision | | |
| 4 | Body | Wide excision | | Primary closure | | | | |
| 5 | Body | Wide excision | Left rectus abdominis flap | SSG | 1993<br><br>1994 | Radical re-excision | | |
| 6 | Body | Subtotal sternectomy | | Primary closure | | | | |
| 7 | Manubrium | Wide excision | Mercilene mesh | Primary closure | | | | |
| 8 | Manubrium | Wide excision | Mesh–PMMA sandwich | Primary closure | | | Lung | Chemotherapy 1999 |

* Performed elsewhere.
SSG spilt skin graft.

Surgical resection was in the form of wide excision[1] in five of the eight patients, including all of those in the past 14 years. Resection was less radical during the earlier years (two of the three patients developing local recurrence subsequently.

Following resection, three patients required thoracic cage reconstruction, one with a metal implant (this patient subsequently had the metal plate removed for infection), one with a mesh and one with a mesh–PMMA sandwich. The mesh and mesh–PMMA sandwich were secured with trans-osseous non-absorbable sutures. Wide resection of the manubrium necessitated bony reconstruction, whereas resection of the body at most necessitated flap reconstruction (pectoralis major advancement or a transverse rectus abdominis musculocutaneous (TRAM) flap). Soft tissue closure is detailed in Table 2.

No patient in our series required ventilatory support (although facilities for this should be available).

Review of operative and pathological reports revealed excision to be complete in patients 3–8 (for patient 1 the records are incomplete and patient 2 had the initial surgical procedure elsewhere). The margins were less than 1 mm in patient 3, and 1 cm in patient 8. In patients 5 and 6, the tumour extended to, but did not breach the pericardium, and in patient 7 the tumour was adherent to the thymic bed, from which it was lifted off.

At the time of writing, three patients are alive and disease free, one is alive with recurrence and four have died, two from their tumour, two from unrelated causes. Of the four patients who are still alive, three have developed recurrences or local metastases (lung, chest and diaphragm). No patient developed distant metastases. The two patients whose deaths were disease related died from local recurrence.

Three patients have had further surgery for local recurrence, consisting of radical re-excision, and in the two patients who are still alive to date there is no evidence of further recurrence.

### Discussion

Primary malignant tumours of the sternum are rare, and accounted for just nine of the 2004 (0.45%) of the primary bone tumours in the Leeds Bone Tumour Registry.[8]

Chondrosarcoma is the most common malignant bone tumour of the sternum, [3,6,8–11]. Chondrosarcomas may arise *de novo* from normal bone or from a pre-existing benign tumour. Only one patient in our series had a tumour arising in a benign tumour (patient 7) and this frequency is similar to other series.[12]

The London Bone and Soft Tissue Tumour Service presents a relatively large series of chondrosarcomas of the sternum, a rare tumour site, covering the last four decades. Our series also confirms the male preponderance demonstrated in previous series[3,6,9–11,13,14].

There are a multitude of causes of a localised swelling of the sternum including infection, inflammation and benign and malignant tumours.[4,15,16] Differentiating between benign chondral lesions and low-grade chondrosarcoma is notoriously difficult, even for a skilled pathologist. Malignant tumours are most commonly metastatic (classically from lung, breast, thyroid or kidney in an adult and a neuroblastoma in children).

Classically, chondrosarcoma presents with persistent pain, especially at night, although in our series of patients a mass was common. Similarly, in the series from the Mayo Clinic[2] and Nottingham,[3] the presenting complaint was most frequently a painful mass. In spite of the fact that the sternum is relatively subcutaneous, there is still a delay from onset of symptoms to diagnosis, similar to other sites of chondrosarcomata.[12]

We feel appropriate management of a swelling of the sternum is as follows. The lesion should be diagnosed by history and examination, followed by plain radiographs (Fig. 1), then accurately staged by magnetic resonance imaging (Fig. 2), computerised tomography of the chest and isotope bone scanning to identify possible osseous metastases. Accurate

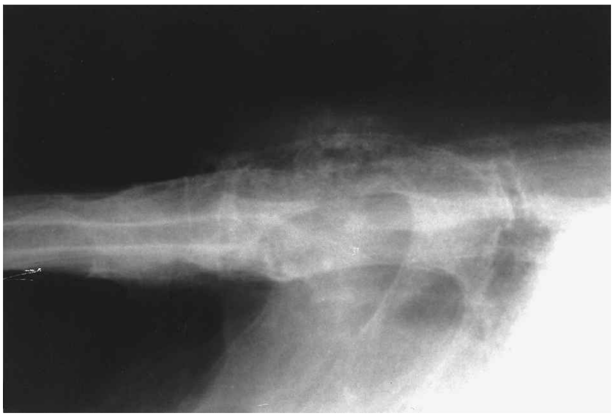

**Fig. 1.** *Lateral radiograph of the sternum demonstrating a mass with cortical disruption.*

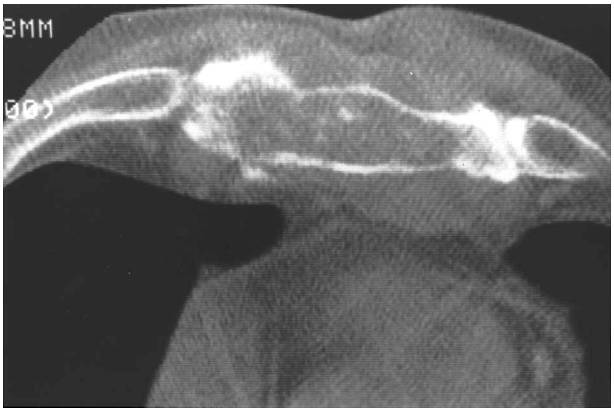

**Fig. 2.** *Magnetic resonance image of a chondrosarcoma of the sternum.*

radiological assessment is necessary as the mass evident on examination may represent only a small part of a much larger tumour invading the sternum. Plain radiographs classically demonstrate a large, lobulated mass with cortical destruction and mottled calcification. It has been stated in previous studies that incision[17] or excision[3,10,11] biopsy be performed in preference to needle biopsy of the tumour. We advocate needle biopsy, with review of the histopathological specimens by an experienced bone tumour pathologist,[4,18]

Preoperative planning of the surgical procedure is essential. Plastic and thoracic surgical assistance should be available if the resection to be performed is extensive. Under antibiotic cover, the sternum is approached via an elliptical incision. The tumour mass is excised en bloc. Where the tumour is in the body of the sternum, dissection is commenced at the xiphoid process and proceeds proximally. Where possible part of the sternum is preserved to maintain the circumferential continuity of the thoracic cage. If the tumour is confined to the manubrium or body, the uninvolved end of the sternum can be preserved, enhancing stability of the chest. For the resection to be considered an adequate wide local excision, a 2.5-cm tumour-free margin[6,21] should be achieved both in bone and costal cartilage. The internal mammary arteries may require ligation.

Reconstruction depends on the site of the tumour: where the defect is in the body and small, soft tissue reconstruction is all that is required. For larger defects there are several methods of reconstruction: using prolene mesh, metal implants or combined mesh and methyl-methacrylate bone cement.[5–7]

Haemostasis is secured and suction drainage used. Primary skin closure is usually possible, but where it is not flap or skin grafting procedures can be utilised. In our series, both pectoralis major advancement and TRAM flaps were utilised. The other flap often used[5] is a latissimus dorsi muscle flap. Pectoralis major advancement and latissimus dorsi muscle flaps have the dual advantages of their blood supplies being more reliable and the muscle being more robust.[5] Appropriate regular follow-up includes clinical examination, chest and lateral sternum radiographs and further staging investigations as necessary.

Chondrosarcomas are difficult to manage, as adequacy of treatment is an important determinant in the incidence of recurrence and duration of disease-free survival. The size of the tumour is an important prognostic feature, determining resectability (even though reconstructive techniques now enable much larger resections to be performed). Chondrosarcomas are resistant to radiotherapy and chemotherapy[6,11] and treatment depends on complete surgical excision of both the tumour (resulting in better long-term survival than limited local excision) and the biopsy tract, without entering the tumour. If the tumour is inadequately resected then it has been shown that there is a high incidence of recurrence (69% compared with 6% in adequately treated patients[12]). We consider adequate surgery to be non-contaminated wide excision. Inadequate procedures are intralesional surgery, marginal excision and any contaminated procedure.[12] Excision of the biopsy tract/scar is essential if surgery is to be considered adequate. Our 5- and 10-year overall survival rates of 57 and 50% are similar to those reported in previous studies,[6,11] although half the deaths in our series were from an unrelated cause in the more elderly patients. Repeated excision of local recurrences are again shown to be worthwhile,[3,10] as illustrated by patient 3 who has had two re-excisions performed and is currently free of disease. Metastatectomy can also result in extended disease-free survival, as illustrated by patient 2 who has had two resections of metastases and is currently alive and disease free.

The margin of resection is important. We advocate a 2.5-cm margin where possible. Of the four patients in our series with small disease free margins (3, 5, 6, 8), three have developed local recurrence or metastatic disease and the fourth has died of the disease.

Wide resection of a tumour of the sternum can be difficult, because the sternum forms a vital link in the integrity of the chest wall. Total sternectomy has a number of associated problems including chest wall instability, exposure of thoracic organs and possible paradoxical respiration. Reconstruction accomplished using one of the options discussed earlier prevents respiratory deficit and protects mediastinal structures.[19] Maintaining the circumferential continuity of the chest avoids paradoxical motion of the chest.

The most important prognostic features in chondrosarcoma are histology (low-grade, well-differentiated tumours have the best prognosis), site of origin (tumours of the appendicular skeleton do better than those of the axial skeleton), adequacy of surgical resection and presenting symptoms (patients presenting with a mass do better than those presenting with pain).[20]

We conclude, therefore, that in chondrosarcoma of the sternum, the single most important factor determining good outcome is a complete resection of the tumour at the initial surgical procedure. This has been shown to reduce the incidence of local recurrence. We also feel that, following staging investigations, biopsy and definitive surgery for this rare tumour site should be performed at a specialist referral centre. As with the management of all musculoskeletal tumours we advocate a team approach. In addition to the orthopaedic oncologist, the particular surgical challenges of a primary sternal tumour lend themselves to the involvement of both plastic and thoracic surgeons in the team.

### Acknowledgements

The authors would like to thank Mrs P.M. Barker, Miss T.H. Tarr, Miss V. Robinson, Ms K. Sharpe, Mr H.B.S. Kemp, Mr J.P. Cobb and Dr D.J. Stoker of The London Bone Tumour Service, Mr E.R. Townsend, Consultant Thoracic Surgeon, Harefield Hospital and Dr I.P. Hopper, Consultant Histopathologist, Derbyshire Royal Infirmary for their help in the preparation of this paper.

### References

1. Enneking WF. A system of staging musculoskeletal neoplasms. *Clin Orthop* 1986; 204: 9–24.
2. King RM, Pairolero PC, Trastek VF, Piehler JM, Payne WS, Bernatz PE. Primary chest wall tumors: factors affecting survival. *Ann Thorac Surg* 1986; 41(6): 597–601.
3. Sabanathan S, Salama FD, Morgan WE, Harvey JA. Primary chest wall tumors. *Ann Thorac Surg* 1985; 39(1): 4–15.
4. Stoker DJ, Cobb JP, Pringle JA. Needle biopsy of musculoskeletal lesions. A review of 208 procedures. *J Bone Joint Surg Br* 1991; 73(3): 498–500.
5. Arnold PG, Pairolero PC. Chest-wall reconstruction: an account of 500 consecutive patients. *Plast Reconstr Surg* 1996; 98(5): 804–10.
6. Martini N, Huvos AG, Burt ME, Heelan RT, Bains MS, McCormack PM, Rusch VW, Weber M, Downey RJ, Ginsberg RJ. Predictors of survival in malignant tumors of the sternum. *J Thorac Cardiovasc Surg* 1996; 111(1): 96–105.
7. Van Schil PE, Van Look R, Van Calster EL, Van Oosterom AT, Hauben EI. Sternal resection for primary presternal and retrosternal mediastinal liposarcoma. *Eur J Cardiothorac Surg* 1996; 10(3): 217–9.
8. Waller DA, Newman RJ. Primary bone tumours of the thoracic skeleton: an audit of the Leeds regional bone tumour registry. *Thorax* 1990; 45(11): 850–5.
9. Eng J, Sabanathan S, Pradhan GN, Mearns AJ. Primary bony chest wall tumours. *J R Coll Surg Edinburgh* 1990; 35(1): 44–7.
10. Sabanathan S, Shah R, Mearns AJ. Surgical treatment of primary malignant chest wall tumours. *Eur J Cardiothorac Surg* 1997; 11(6): 1011–6.
11. McAfee MK, Pairolero PC, Bergstralh EJ, Piehler JM, Unni KK, McLeod RA, Bernatz PE, Payne WS. Chondrosarcoma of the chest wall: factors affecting survival. *Ann Thorac Surg* 1985; 40(6): 535–41.
12. Gitelis S, Bertoni F, Picci P, Campanacci M. Chondrosarcoma of bone. The experience at the Istituto Ortopedico Rizzoli. *J Bone Joint Surg Am* 1981; 63(8), 1248-1257.
13. Graeber GM. Chest wall resection and reconstruction. *Semin Thorac Cardiovasc Surg* 1999; 11(3): 251–63.
14. Evans HL, Ayala AG, Romsdahl MM. Prognostic factors in chondrosarcoma of bone: a clinicopathologic analysis with emphasis on histologic grading. *Cancer* 1977; 40(2): 818–31.
15. Dahlin DC, Henderson ED. Chondrosarcoma, A surgical and pathological problem. *J Bone Joint Surg Am* 1956; 38: 1025–38.
16. Hart FD. *French's Index of Differential Diagnoses*. Bristol: J Wright & Son, 1985.
17. McCormack P, Bains MS, Beattie EJ Jr, Martini N. New trends in skeletal reconstruction after resection of chest wall tumors. *Ann Thorac Surg* 1981; 31(1): 45–52.
18. Pringle JA. Pathology of bone tumours. *Balliere's Clin Oncol* 1996; 1: 223–41.
19. Gabbay S, Bennett RD, Amato J, Cherny EJ. Controversies in management of sternal tumors. *Ann Thorac Surg* 1989; 48(3): 428–31.
20. Harwood AR, Krajbich JI, Fornasier VL. Radiotherapy of chondrosarcoma of bone. *Cancer* 1980; 45(11): 2769–77.
21. Mansour KA, Anderson TM, Hester TR. Sternum resection and reconstruction. *Ann Thorac Surg* 1993; 55: 838–43.
